# Take a break: should breaks be enforced during digital breast tomosynthesis reading sessions?

**DOI:** 10.1007/s00330-023-10086-4

**Published:** 2023-08-17

**Authors:** George John William Partridge, Adnan Gani Taib, Peter Phillips, Jonathan Jeffrey James, Keshthra Satchithananda, Nisha Sharma, Juliet Morel, Rita McAvinchey, Alexandra Valencia, William Teh, Humaira Khan, Elizabeth Muscat, Michael James Michell, Yan Chen

**Affiliations:** 1 Translational Medical Sciences, School of Medicine, University of Nottingham, Clinical Sciences Building, City Hospital Campus, Hucknall Road, Nottingham, NG5 1PB UK; 2 Health and Medical Sciences Group, University of Cumbria, Lancaster, UK; 3 Nottingham University Hospitals NHS Trust, Nottingham Breast Institute, City Hospital Campus, Hucknall Road, Nottingham, NG5 1PB UK; 4 Department of Breast Radiology and National Breast Screening Training Centre, King’s College Hospital, Denmark Hill, London, SE5 9RS UK; 5 Leeds Breast Screening Unit, Leeds Teaching Hospital, York Road, Leeds, LS14 6UH UK; 6 Jarvis Breast Screening Centre, Guildford, Surrey, GU1 1LJ UK; 7 Avon Breast Screening, Bristol Breast Care Centre, Bristol, BS10 5NB UK; 8 North London Breast Screening Service, Edgware Community Hospital, London, HA8 9BA UK; 9 City, Sandwell and Walsall Breast Screening Service, Birmingham City Hospital, B18 7QH, Birmingham, UK; 10 South West London Breast Screening Service, St George’s Hospital, London, SW17 0QT UK

**Keywords:** Mammography, Digital breast tomosynthesis (DBT), Fatigue, Eye tracking technology, Blinking

## Abstract

**Objectives:**

Digital breast tomosynthesis (DBT) can improve diagnostic accuracy compared to 2D mammography, but DBT reporting is time-consuming and potentially more fatiguing. Changes in diagnostic accuracy and subjective and objective fatigue were evaluated over a DBT reporting session, and the impact of taking a reporting break was assessed.

**Materials and methods:**

Forty-five National Health Service (NHS) mammography readers from 6 hospitals read a cancer-enriched set of 40 DBT cases whilst eye tracked in this prospective cohort study, from December 2020 to April 2022. Eye-blink metrics were assessed as objective fatigue measures. Twenty-one readers had a reporting break, 24 did not. Subjective fatigue questionnaires were completed before and after the session. Diagnostic accuracy and subjective and objective fatigue measures were compared between the cohorts using parametric and non-parametric significance testing.

**Results:**

Readers had on average 10 years post-training breast screening experience and took just under 2 h (105.8 min) to report all cases. Readers without a break reported greater levels of subjective fatigue (44% vs. 33%, *p *= 0.04), which related to greater objective fatigue: an increased average blink duration (296 ms vs. 286 ms, *p *< 0.001) and a reduced eye-opening velocity (76 mm/s vs. 82 mm/s, *p *< 0.001). Objective fatigue increased as the trial progressed for the no break cohort only (*p*s < 0.001). No difference was identified in diagnostic accuracy between the groups (accuracy: 87% vs. 87%, *p *= 0.92).

**Conclusions:**

Implementing a break during a 2-h DBT reporting session resulted in lower levels of subjective and objective fatigue. Breaks did not impact diagnostic accuracy, which may be related to the extensive experience of the readers.

**Clinical relevance statement:**

DBT is being incorporated into many mammography screening programmes. Recognising that reporting breaks are required when reading large volumes of DBT studies ensures this can be factored in when setting up reading sessions.

**Trial registration:**

Clinical trials registration number: NCT03733106

**Key Points:**

*• Use of digital breast tomosynthesis (DBT) in breast screening programmes can cause significant reader fatigue.*

*• The effectiveness of incorporating reading breaks into DBT reporting sessions, to reduce mammography reader fatigue, was investigated using eye tracking.*

*• Integrating breaks into DBT reporting sessions reduced reader fatigue; however, diagnostic accuracy was unaffected.*

**Supplementary Information:**

The online version contains supplementary material available at 10.1007/s00330-023-10086-4.

## Introduction

Adopting digital breast tomosynthesis (DBT) as a standard of care in breast screening programmes could improve patient outcomes and clinical workflow due to its reported increase in cancer detection rate and reduced recall rate in high recall environments, compared to 2D digital mammography alone [1–5]. DBT facilitates cancer detection by offering greater power to resolve overlapping layers of breast tissue, reducing the likelihood of missing obscured lesions or recalling artefactual findings. As a consequence, DBT images present greater image content to search and interpret compared to 2D digital mammography, increasing the read time and cognitive cost to the clinician [6]. In the context of breast screening, reading large volumes of DBT images could induce more severe fatigue in mammography readers compared to 2D studies, which has the potential to compromise diagnostic accuracy over prolonged screening sessions.

Previous studies in radiology have demonstrated the negative effects of reader fatigue on diagnostic accuracy and case interpretation efficiency [7–9]. However, these studies have primarily focused on specialties and imaging modalities other than DBT. Furthermore, these studies often compare radiologists’ performance in two different reporting sessions when fatigue levels would be expected to be very different, for instance, comparing a reporting session before starting a work shift to one after finishing a shift and comparing reporting in day shifts to overnight shifts [8, 9].

The aim of this prospective cohort study was to evaluate the changes in diagnostic accuracy and subjective and objective fatigue over a DBT reporting session, and how taking a break in reporting can affect these parameters. We hypothesised that implementing breaks within a DBT session would lead to lower levels of fatigue and reduced error rates. Identifying the point of fatigue onset in DBT reporting via blink characteristics could help to inform standards of DBT reporting session duration to limit reader fatigue and its negative impacts on patient outcomes, as breast screening programmes transition to this new modality.

## Materials and methods

### Participants and inclusion criteria

This prospective cohort study was conducted as a sub-study within the UK PROSPECTS Trial (ClinicalTrials.gov Identifier: NCT03733106) which has London–Dulwich Research Ethics Committee approval and all study participants provided written consent. The PROSPECTS Trial is a prospective randomised trial of DBT plus standard 2D digital mammography or synthetic 2D mammography (S2D) compared to standard 2D digital mammography in breast cancer screening.

Forty-five mammography readers from 6 National Health Service Breast Screening Programme centres participated from December 2020 to April 2022. All readers from centres participating in the PROSPECTS Trial were invited to take part, and consenting readers were consecutively recruited at each centre. Participants were NHS Breast Screening Programme mammography readers including board-certified consultant radiologists, radiographers (consultant radiographers and advanced practitioners, who are technologists with Master’s level training in mammographic interpretation) and breast clinicians (doctors who work in the field of breast care, but are not radiologists). All screening mammograms in the NHS breast screening programme are independently double read; and all participating mammography readers interpreted a minimum of 5000 mammograms per year, with a minimum of 1500 screening mammograms as the first reader. All readers had received prior training in DBT interpretation. Participants received continuing professional development (CPD) points and certification for their participation.

Eye tracking data from 30 of the 45 participants included in the present investigation were analysed previously [10, 11]. These studies investigated the use of eye-blink behaviour as fatigue and cognitive markers in DBT reporting; but 21 of these 30 participants had reporting breaks in their reading sessions—a previous limitation [10, 11]. In the present study, data has been collected from a further 15 participants who were not permitted a reporting break, enabling a comparison between participants depending on whether a break was allowed.

### DBT case set

Participants independently read 40 anonymised cases possessing both 2D digital mammography and DBT images. Cases were chosen by an expert breast radiologist with more than 20 years’ experience, J.J., providing a variety of difficulty. There was also a variety of case pathology and finding types (Table 1). Participants were blinded to the proportions of each pathology type in the test set. Cases were presented to each participant in a random order. Case images were viewed on a Hologic SecurView workstation (Hologic Inc.) with a 4200 × 2800 pixel, mammography-approved BARCO monitor (BARCO Ltd.). Up to 4 views (left and right breast, MLO and CC) from a single case could be reviewed. Cases opened with 2D digital mammography images by default. DBT mode could be toggled on and off, reflecting real clinical practice (note that participants preferentially read the cases in DBT mode). The hanging protocols for each case could be changed by the participant, and all image manipulation tools were available to allow participants to simulate real-life reading.

**Table 1:** DBT case information

	Frequency (*n*)
Breast pathological outcome	
Normal	16
Benign	5
Malignant	19
Radiological feature types of malignant lesions	
Architectural distortion	1
Asymmetry	1
Calcification	3
Ill-defined mass	4
Spiculated mass	9
Well-defined mass	1
Breast density (%)	
≤ 25	13
25 < density ≤ 50	17
51 < density ≤ 75	9
≥ 75	1
Case difficulty (judged by expert panel)	
Very easy	4
Easy	15
Difficult	20
Very difficult	1

### Eye tracking equipment

**Fig. 1:**
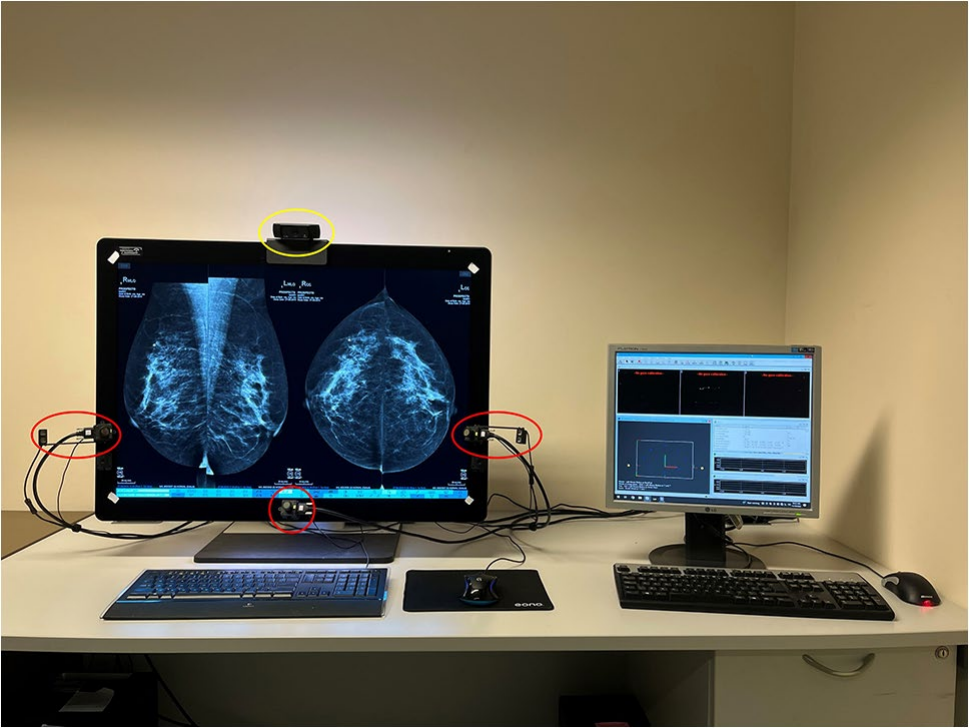
Experimental setup. Eye tracking cameras (red circles) and a scene camera (yellow circle) positioned on a participant workstation. The monitor to the right was used for eye tracking calibration and monitoring. This was not visible to the participant during the experiment. During the experiment, the lights were dimmed

Three non-intrusive eye tracking cameras (SmartEyePro, SmartEye AB) were mounted to the clinical monitor to record eye-blink data (60 Hz sampling rate). Equipment was set up in each participant’s natural reading environment, at their NHS screening centre (Fig. 1).

### Procedure

Before the experiment, participants completed a demographics survey to account for confounding variables and read two practice cases to ensure familiarity with the procedure and image viewing software (these were not recorded). Participants then examined each case in their own time and verbally reported each breast as normal or benign (return to screen) or indeterminate, suspicious or highly suspicious (recall). Participants indicated the location of any abnormality on the images, which were recorded using the PERFORMS online reporting software by a supervising experimenter [12, 13]. PERFORMS (Personal Performance in Mammographic Screening) is an international external quality assurance scheme for mammography readers; further details on PERFORMS can be found elsewhere [14].

For the first cohort of eye tracking trials (*n* = 20; 3 screening centres), participants had reporting breaks every 40 min. The duration of the break was measured, but we did not record what the reader did during this period. For the second cohort (*n *= 24; the remaining 3 centres), participants were not permitted to take a break. One extra participant from the second batch needed to take a break at 40 min, therefore was categorised as having a break (*n *= 1). Participants completed a subjective fatigue survey before and after the reporting session, where participants rated their fatigue levels on a percentage scale from 0 (not fatigued) to 100 (extremely fatigued).

### Blink data processing, quality filtering and exclusion criteria

**Fig. 2:**
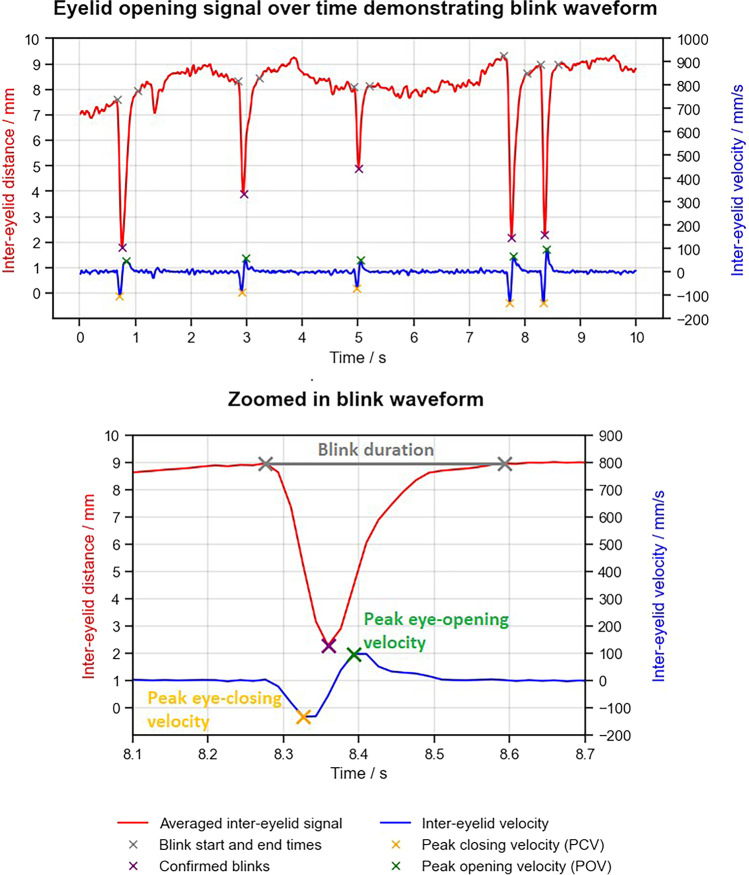
Plots to demonstrate how blink metrics were calculated from the inter-eyelid distance, obtained from eye tracking. Top plot shows a 10-s clip of a participant’s inter-eyelid distance (red), and the calculated inter-eyelid velocity (blue), containing five blinks. Eye-blink events are identifiable by a rapid, large-magnitude reduction in inter-eyelid distance, followed by a rapid increase in inter-eyelid distance back to the eye open level. Smaller fluctuations in inter-eyelid signal when the eye is open are a consequence of gaze-related partial eyelid closures. The fifth blink in the top plot is isolated and shown in greater detail in the lower plot. Key features of the blink are annotated, noting the blink duration (grey) and the peak eye-opening velocity (green), which are assessed as objective fatigue metrics in the present study

Studies in a variety of psychological settings have reported increased blink rates in the fatigued state, as well as changes in individual blink dynamics, including longer blink durations in the fatigued state [15–19]. Previous investigation during DBT reporting concluded that blink frequency was an unreliable measure of fatigue in this context, and hence, only characteristics of the blink events (including blink duration and peak eye-opening velocity Fig. 2) were analysed here [10, 11].

**Fig. 3:**
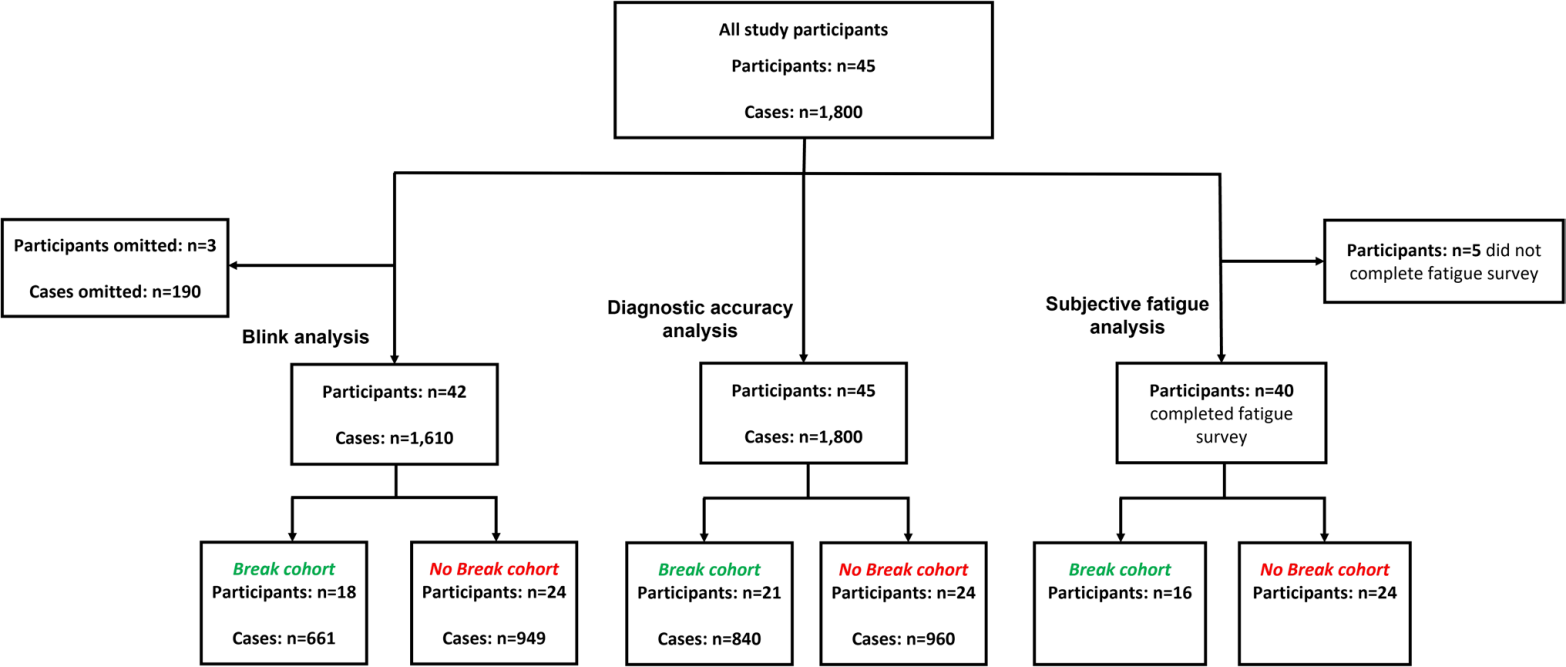
Flow chart demonstrating data exclusion and quality filtering for each analysis

Eye-blink data were analysed using a blink detection algorithm developed in-house [20, 21]. Blink data were automatically subjected to quality assessment as part of the algorithm; cases that did not pass the quality filter, due to missing and noisy data (resulting from eye obstruction and calibration issues), were excluded (Fig. 3).

### Statistical analysis

All DBT cases had a known outcome. Malignant and benign cases were confirmed by biopsy, and normal cases had a normal 3-year follow-up mammogram. For each participant, each case was classified as true positive (TP), true negative (TN), false positive (FP) or false negative (FN) compared to pathology.

Normality was tested for using the Kolmogorov-Smirnov test. Significance testing was calculated for the differences between the break and no break cohorts. A chi-square test of independence was performed to examine the relation between categorical variables. The Mann-Whitney *U* test and independent samples *t*-test were used to check for significance for non-parametric and parametric continuous variables respectively. Kruskal-Wallis tests were performed to investigate the change in non-parametric blink metrics over the course of the DBT reporting session. The *α*-level for statistical significance was set at .05 for all analyses. Statistical calculations were performed using Python 3.8.3 (Python Software Foundation) by GP and AT. Data generated or analysed during the study are available from the corresponding author by request.

## Results

### Participant characteristics

We initially included 45 participants who reported 40 DBT images, yielding 1800 cases (Fig. 3). Following quality filtering, the blink data associated with 190 cases were excluded due to poor-quality eye tracking data. Three participants were excluded since all of their associated cases were excluded. Forty-two participants with 1610 cases remained in the blink analysis. All 45 participants remained in the diagnostic accuracy analysis. Forty participants were included in the subjective fatigue analysis; 5 were excluded due to an incomplete fatigue survey.

Demographic, training and session duration information for the participants are illustrated in Table 2. Most readers were radiology consultants (69%, *n *= 31 of 45) followed by advanced radiographic practitioners (16%, *n *= 7 of 45). We found no evidence of a difference in job roles between the break and no break cohorts (*p *= 0.54). Similarly, there was no significant difference between the cohorts in the frequencies of corrective lenses, day of the week and time of session (*p *= 0.37, *p *= 0.11, *p *= 0.66, respectively). The break cohort consisted mainly of readers from earlier sites, whereas the no break cohort were mainly from later sites (*p *< 0.001)—reflecting the change in methodology mentioned previously.

**Table 2:** Participant demographics, experience and trial timing. *SD* standard deviation, *IQR* interquartile range

Characteristic	All participants (*n *= 45)	Break cohort (*n *= 21)	No break cohort (*n *= 24)	*p* value
Gender, female, *n* (%)	39 (87)	16 (76)	23 (96)	.05
Job role, *n* (%)				.54
Radiology consultant Advanced practitioner Consultant radiographer Breast surgeon	31 (69) 7 (16) 5 (11) 2 (4)	16 (76) 3 (14) 2 (10) 0 (0)	15 (63) 4 17) 3 (13) 2 (8)	
Screening centre, *n* (%)				< .001
1 2 3 4 5 6	4 (9) 5 (11) 11 (24) 10 (22) 5 (11) 10 (22)	4 (19) 5 (24) 11 (52) 0 (0) 0 (0) 1 (5)	0 (0) 0 (0) 0 (0) 10 (42) 5 (21) 9 (38)	
Corrective lenses, *n* (%)				.37
None Glasses Contact lenses	18 (40) 20 (44) 7 (16)	10 (48) 7 (33) 4 (19)	8 (33) 13 (54) 3 (13)	
Day of the week, *n* (%)				.11
Monday Tuesday Wednesday Thursday Friday	9 (20) 7 (16) 9 (20) 10 (22) 10 (22)	7 (33) 3 (14) 2 (10) 6 (29) 3 (14)	2 (8) 4 (17) 7 (29) 4 (17) 7 (29)	
Time of trial, *n* (%)				.66
Morning Afternoon or evening	22 (49) 23 (51)	11 (52) 10 (48)	11 (46) 13 (54)	
Years in post, median (IQR)	10 (12)	9.0 (11)	10.5 (14)	.43
Years of DBT reading experience, median (IQR)	5 (4)	5.0 (4)	5.5 (5)	.44
Number of DBT cases read/year, median (IQR)	500 (775)	400 (1025)	500 (825)	.86
Duration of trial excluding breaks, mean minutes (SD)	105.8 (29)	109.9 (31)	102.0 (28)	.38
Duration of breaks, median (IQR)	-	7.6 (9)	-	-

Both cohorts were well matched in terms of experience. We found no evidence of a difference in the years in post, years reading DBT and number of DBT cases read per year (*p *= 0.43, *p *= 0.44, *p *= 0.86, respectively). Participants had a sound baseline experience in radiology illustrated by a median of 10 years in their post (IQR=12 years). The median DBT experience was 5 years (IQR = 5 years), suggesting participants were not novices in DBT interpretation.

Participants on average took just under 2 h to complete reading all 40 DBT cases. Session duration (excluding break durations) was similar between the two cohorts (109.9 min vs. 102.0 min, *p *= 0.38). The median duration of a break was 7.6 min (IQR = 9 min).

### Subjective fatigue

**Fig. 4:**
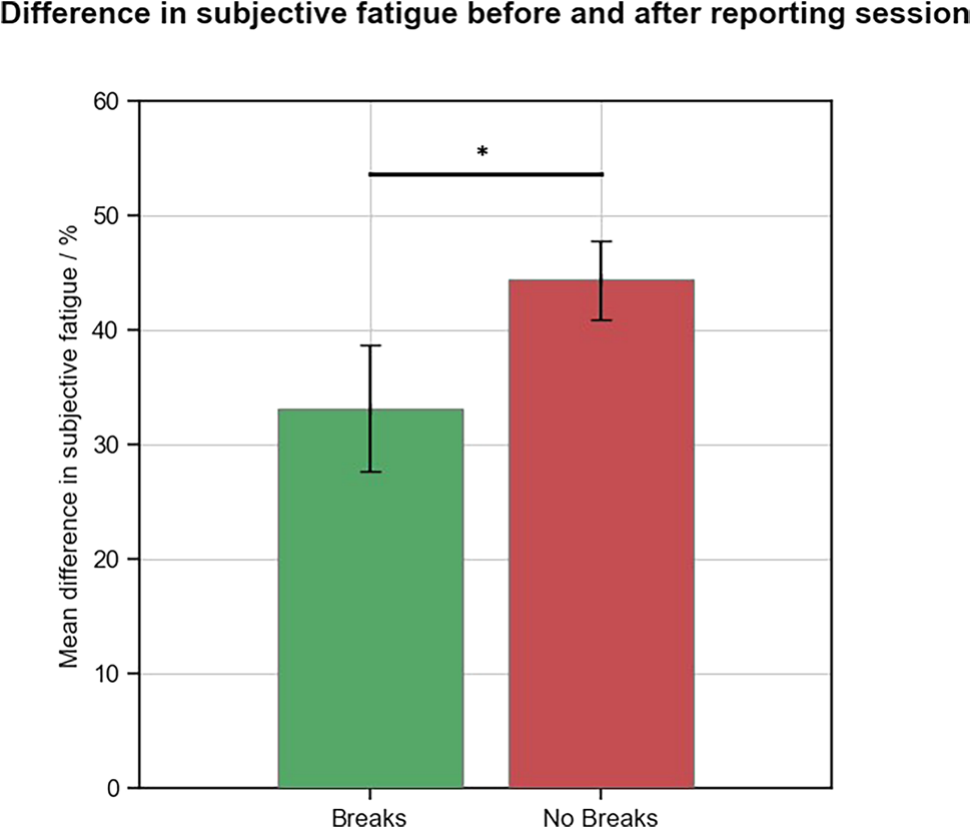
Bar chart illustrating differences in subjective fatigue levels between participants with and without breaks; error bars represent the standard error of the mean. Cohorts were compared using a Mann-Whitney *U* test, *Significance *p *< 0.05

Of the participants who completed the fatigue survey, those who had a break (*n* = 16) reported significantly lower levels of subjective fatigue difference after the reading sessions compared to those who did not have breaks (*n *= 24) (mean, SD: 33% ± 22 vs. 44% ± 17, respectively, *p *= 0.04; Fig. 4). We found no evidence of a difference in the starting levels of fatigue between the break and no break cohorts (mean, SD: 32% ± 0.2 vs. 24% ± 0.2, respectively, *p *= 0.19).

### Objective fatigue (blink metrics)

**Fig. 5:**
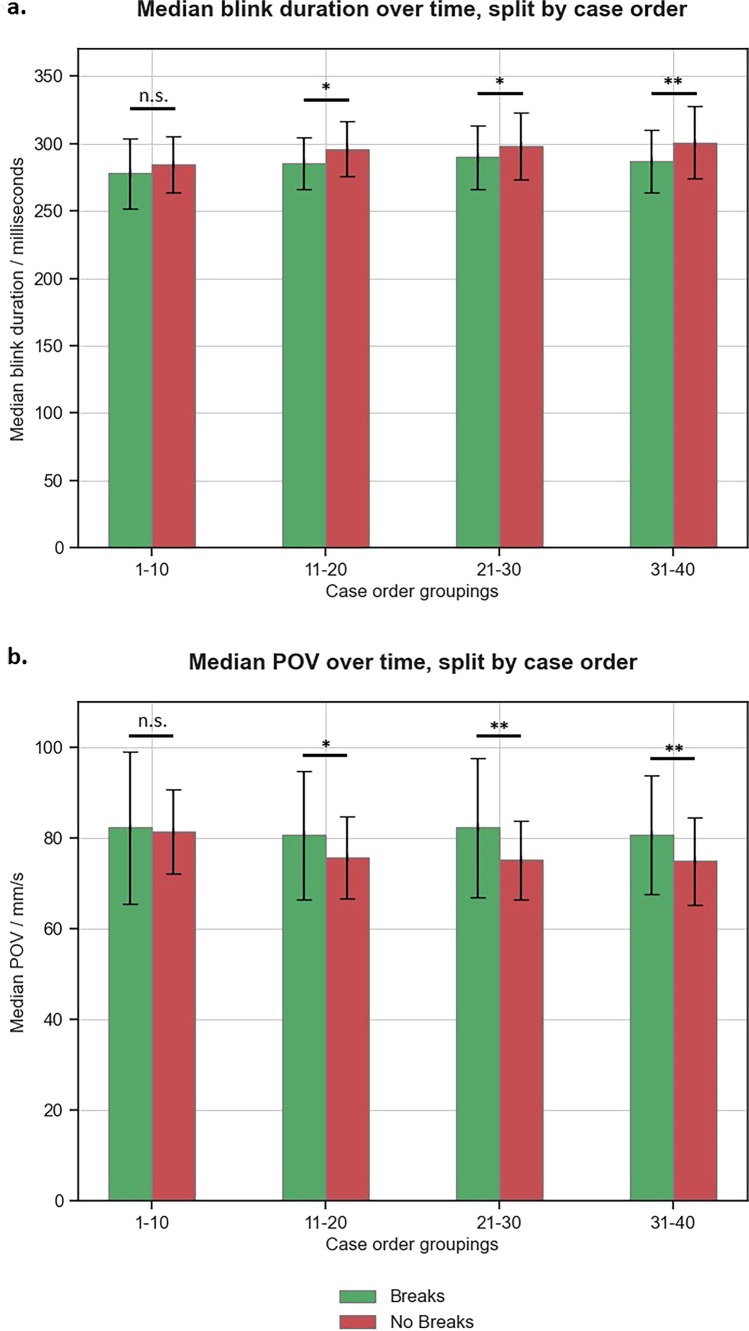
Bar charts illustrating the change in the blink metrics of the break (green) and no break (red) cohorts over time in the reporting session (**a** blink duration, and (**b**) peak eye-opening velocity [POV]). Blink data from each cohort were divided into bins of ten cases. Error bars represent IQR. In each case order group, blink data were compared by Mann-Whitney *U* tests; n. s. (no significance) denotes *p *> 0.05, * denotes significance *p *< 0.05, ** denotes significance *p *< 0.005

Over the whole trial, participants in the no break cohort exhibited a greater blink duration (296 ms vs 286 ms, *p *< 0.001), and a reduced peak eye-opening velocity (POV) (76 mm/s vs 82 mm/s, *p *< 0.001), compared to the break cohort. Additionally, changes in blink metrics in both cohorts were noted over the time course of the reporting session, where the session was split by case chronology (Fig. 5). During the interpretation of the first 10 cases, the blink metrics were similar between the two cohorts (blink duration: 285 ms vs 282 ms, *p *= 0.14; POV: 81 mm/s vs 82 mm/s, *p *= 0.19). However, during the second, third and last 10 cases, the blink duration was greater in the no break cohort (*p *= 0.02, *p *= 0.01 and *p *= 0.003, respectively), and the POV was reduced in the no break cohort (*p *= 0.02, *p *< 0.001 and *p *< 0.001, respectively) compared to the break cohort.

Using Kruskal-Wallis tests to compare blink metrics in each cohort over the time course revealed no evidence of a difference between case order and blink duration or POV in the break cohort (*p *= 0.09 and *p *= 0.88, respectively). However, significant changes were noted in the no break cohort (*p *< 0.001 and *p *< 0.001). Post hoc pairwise tests in the no break cohort highlighted significant changes in the blink metrics after reporting the first 10 cases compared to later cases (Supplementary Tables 1 and 2).

### Diagnostic accuracy

All participants (*n *= 45) had a median sensitivity of 94.7% (IQR = 10.5%), a mean specificity of 85.1% (SD = 7.5%) and a mean accuracy of 87.1% (SD = 5.4%). There was no evidence of a difference in the three diagnostic accuracy measures investigated between the break and the no break groups (Table 3). Additionally, the diagnostic accuracy measures were similar for both groups when the reading session was split by case chronology (Table 3). Although not significant, a 10% reduction was observed for sensitivity in the no break cohort when comparing the performance in the first 20 cases to the second 20 cases (100% vs. 90%, *p *= 0.09), whereas the sensitivities in the break cohort were matched when the session was divided in this way (92.3% vs. 92.3%, *p *= 0.27).

**Table 3:** Comparison of diagnostic accuracy between cohorts. Bolded values relate to direct comparison between the break and no break cohort. Italicised values relate to comparison within break and no break cohorts based on case order. ^a^Median (IQR); ^b^Mean (standard deviation)

Diagnostic accuracy	Break (*n* = 21 participants)	No break (*n* = 24 participants)	*p* value
Sensitivity, % *Cases 1–20* *Cases 21–40* *p value for chronology*	**94.7 (8.8)** ^a^ *92.3 (10.6)* *92.3 (15.6)* *.27*	**94.7 (5.5)** ^a^ *100.0 (10.0)* *90.0 (12.5)* *.09*	***p *= .36**
Specificity, % *Cases 1–20* *Cases 21–40* *p value for chronology*	**85.3 (7.8)** ^b^ *85.2 (6.9)* *85.5 (11.2)* *.92*	**85.0 (7.5)** ^b^ *83.4 (10.3)* *86.9 (9.9)* *.22*	***p *= .89**
Accuracy, % *Cases 1–20* *Cases 21–40* *p value for chronology*	**87.0 (6.0)** ^b^ *87.3 (5.1)* *86.7 (9.5)* *.73*	**87.2 (5.0)** ^b^ *86.6 (7.6)* *87.8 (7.6)* *.60*	***p *= .92**

## Discussion

Digital breast tomosynthesis (DBT) has the potential to transform screening programmes; however, fatigue and its potential negative impacts on diagnostic accuracy need to be considered. In our study, two cohorts of mammography readers read a cancer-enriched set of 40 DBT cases, with and without breaks. Those without a break reported greater levels of subjective fatigue post reporting session (44% vs. 33%, *p *= 0.04) which was related to a greater blink duration and reduced peak eye-opening velocity (POV), compared to those who had breaks (blink duration: 296 ms vs. 286 ms, *p *< 0.001; POV: 76 mm/s vs 82 mm/s, *p *< 0.001). Furthermore, an increase in the blink duration and a reduction in the POV were noted as the trial progressed for the no break cohort (*p *< 0.001 and *p *< 0.001). There was no evidence of a difference in diagnostic accuracy between the cohorts (*p *= 0.92) or over time within either cohort (*p *= 0.73 and *p *= 0.60).

Due to the nature of the study, the first 20 participants were from specific screening centres, whom all had breaks. However, since both cohorts were equally matched in potential confounding factors, we do not expect the differences in centre location as a cause of significant findings. Notably, the readers in this study had extensive radiology experience (including DBT). Ten years was the average time in post, with half of that time reading tomosynthesis cases. This is generalisable to the current screening radiologists in the NHS [22].

Several studies in a diverse number of study environments have demonstrated that blink duration and peak eye-opening velocity are reliable markers of fatigue, noting a positive correlation between blink duration and fatigue, and a negative correlation between POV and fatigue [15–18, 23]. This was reflected in our study where blink duration was greater, and POV reduced in the no break cohort (*p *< 0.001 and *p *< 0.001). Furthermore, blink metrics were similar between both cohorts at the start of the trial (for the first 10 cases) and then increased through the trial in the no break cohort (*p *< 0.001 and *p *< 0.001), whereas the blink metrics were more stable through the trial in the break cohort. The significant changes in the blink metrics over time were most notable between the first and second 10 cases for the no break group. These findings suggest that the fatigue level of the no break participants increased considerably after reporting the first 10 cases and increased more gradually through the remainder of the session. Conversely, the fatigue level of the participants in the break cohort was more consistent through the trial, potentially related to the presence of reading breaks in their reporting sessions. Interestingly, reporting breaks were only relatively short, lasting on average 7.6 min, yet still seemed to have a marked effect on the subjective and objective fatigue measures.

We observed no evidence of difference in diagnostic accuracy between the two cohorts. This may be related to the extensive experience level of the study participants. Bernstein et al [24] recently explored the effect of time of day on DBT interpretation and reported that radiologists with 5 or fewer years of post-training experience exhibited increased recall and false positives with every consecutive hour of DBT reading (with increasing fatigue). However, there was no increased recall or false positives for radiologists with more than 5 years of experience [24]. Krupinski et al [8] investigated the performance of radiology residents and consultants in lung CT nodule detection before starting and after finishing a work shift. Fatigue measures were greater for all participants in the later session, and receiver operating characteristic analyses showed that resident performance reduced in the later session, but consultant performance actually improved from the earlier to later session [8]. These results suggest that experienced radiologists are more resistant to the negative impacts of fatigue than relative novices, and potentially a higher threshold of fatigue is required to elicit a meaningful reduction in diagnostic accuracy for these clinicians [8, 24].

Study limitations should be acknowledged. The test set only contained a relatively small number of cases enriched with challenging cancers and so is not representative of typical screening populations; consequently, reader behaviour may not be generalisable to real-world reporting. Additionally, diagnostic accuracy metrics may have been artificially high due to the Hawthorne effect [25, 26]. In the screening population, only a small number of cases are true positives, and so in a loaded malignant case set, recall behaviour may be exaggerated. Artificially high recall rates may have blunted any potential difference in diagnostic accuracy. Future studies should also include junior mammography readers. These participants will constitute a large proportion of future readers utilising DBT routinely for screening. Therefore, it would be beneficial to understand how fatigue impacts their reporting. Finally, although participants provided subjective fatigue levels using a percentage scale, a validated fatigue questionnaire could have been implemented [27].

In conclusion, a break during a 2-h DBT reporting session resulted in lower levels of subjective fatigue. Blink metrics, recognised as objective fatigue measures, demonstrated a significant increase in fatigue for participants that were not permitted breaks compared to those who were and were seen to increase significantly for participants without reporting breaks as the trial progressed. Implementing breaks did not significantly impact diagnostic accuracy in this study; however, this may be related to the experienced sample of radiologists, case mix and number of cases in the reporting session. With the potential serious, but preventable harm related to fatigue, and the growing uptake of DBT into screening programmes, it is vital to understand how fatigue manifests in mammography readers reporting with this modality. Information from these studies can help to inform clinical guidelines and standards on the optimal length of time or number of cases per reading session before onset of fatigue.

### Supplementary Information

ESM 1 (PDF 170 kb)

## Abbreviations

DBT: Digital breast tomosynthesis
IQR: Interquartile range
NHS: National Health Service
SD: Standard deviation

## Declarations

### Guarantor

The scientific guarantor of this publication is Yan Chen.

### Statistics and biometry

No complex statistical methods were necessary for this paper.

### Study subjects or cohorts overlap

The eye tracking data from 30 of the 45 participants included in the present investigation were analysed previously [1, 2]. These studies investigated the use of eye-blink behaviour as fatigue and cognitive markers in DBT reporting; but 21 of these 30 participants had reporting breaks in their reading sessions—a previous limitation [1, 2]. In the present study, data has been collected from a further 15 participants who were not permitted a reporting break, enabling a comparison between participants depending on whether a break was allowed.

[1]Chen Y, Sudin E, Partridge G, Taib A, Darker I, Phillips P, et al Measuring reader fatigue in the interpretation of screening digital breast tomosynthesis. British Journal of Radiology 2023 Jan 12;96(1143):20220629. doi: 10.1259/bjr.20220629.

[2]Partridge G, Phillips P, Darker I, Chen Y. Investigating reading strategies and eye behaviours associated with high diagnostic performance when reading digital breast tomosynthesis (DBT) images. Medical Imaging 2022: Image Perception, Observer Performance, and Technology Assessment 2022;12035:36–47. 10.1117/12.2611388.

### Methodology

- prospective
- experimental
- multicentre study
