# Supplementary material for: Take a break: should breaks be enforced during digital breast tomosynthesis reading sessions?
Source: Eur Radiol. 2023 Aug 17;34(2):1388–98. doi: 10.1007/s00330-023-10086-4 (PMC10853356; doi:10.1007/s00330-023-10086-4)
Supplement: Supplementary file 1 — (PDF 170 kb) [file 330_2023_10086_MOESM1_ESM.pdf]

Supplementary tables

Case order groupings (no break cohort)	Cases 1-10	Cases 11-20	Cases 21-30	Cases 31-40
**Cases 11-20**	***.02***	-	-	-
**Cases 21-30**	***<.001***	.06	-	-
**Cases 31-40**	***<.001***	.10	.83	-

Supplementary Table 1: Pairwise Mann-Whitney U tests for blink duration between case order groupings in the no break cohort following Kruskal-Wallis test.
Bolded p-values are significant.

Case order groupings (no break cohort)	Cases 1-10	Cases 11-20	Cases 21-30	Cases 31-40
**Cases 11-20**	***.002***	-	-	-
**Cases 21-30**	***<.001***	.49	-	-
**Cases 31-40**	***<.001***	.39	.88	-

Supplementary Table 2: Pairwise Mann-Whitney U tests for POV between case order groupings in the no break cohort following Kruskal-Wallis test.
Bolded p-values are significant.
